# Monthly Summary

**Published:** 1877-07

**Authors:** 


### MONTHLY SUMMARY.

*Precautions in Using the Hypodermic Syringe.*—In the last volume of the *Transactions* of the West Virginia State Medical Society, Dr. John Frissell relates a case of mammary cancer proceeding from the puncture of a hypodermic syringe. He adds the following directions regarding the use of this instrument:—

In the first place the needle is to be polished and washed until it becomes perfectly clean and smooth.

Secondly, after thoroughly cleansing the inside of both needle and syringe, the fluid to be injected is to slowly drawn in.

Then, thoroughly onointing the needle with a solution of carbolized glycerine (glycerine eight drachms, carbolic acid one drachm), the skin at the point selected for the injection is to be seized between the thumb and forefinger of the left hand, lifted up and made tense, and the needle pushed through at right angles, taking special care that its point neither touches muscle, gland, or other solid tissue. The fluid should be then slowly injected into the cellular tissue. After the withdrawal of the needle should be placed over its point of entrance and the part rubbed and pressed until it becomes perfectly smooth and flat.

Should there be any irritating ingredients in the injected fluids their action, of course, must be regarded as independent of the condition of needle and syringe.

When I have been careful to follow strictly the direction, above given, in the use of the hypodermic syringe, I have seen neither sores, tumors, abscesses, nor bad results of any kind follow.—*Med. and Surg. Reporter.*

---

*Death from Chloroform.*—A case of death whilst under the influence of chloroform recently took place at the Derby Infirmary. Deceased, who was fifty-six years of age, was about to undergo an operation for fistula and hemorrhoids, but before he was ready for operation, the respiration suddenly became very irregular, he struggled violently, and the pulse, which had up to this time been good, ceased. In spite of all the means resorted to for a considerable time, he showed no signs of rallying from the first. The quantity of chloroform which had been poured into the lint-holder was in all about three drachms. The *post-morten* examination did not reveal any organic disease.—*Brit. Med. Journal,* March 17, 1877.

---

*Action of Anæsthetics on Muscles and Nerves.*—M. Couly, of Paris, finds that when animals are killed by chloroform, ether, or chloral, the muscles and motor nerves retain their irritability much longer than when death is produced by bleeding, compression of the heart, or asphyxia. This is especially marked in the case of chloral. The author considers the cause of this phenomenon to consist, not in any action of the anæsthetics on the spinal cord, but in a direct modification of the nerves and muscles by them through the blood, similar to that which occurs in poisoning by carbonic oxide.—*Med. and Surg. Rep.*

*Croton-Chloral in Facial Neuralgia.*—DR. F. B. LEE reports the case of a lady, æt. 32, who had suffered for years from attacks of facial neuralgia, had several teeth extracted, had been blistered behind the ears, and had tried numerous other means without avail. He prescribed croton-chloral in three-grain doses every four hours. After the third dose perfect ease was experienced, and although three months had elapsed, there had been no return of the disease.—*The British Med. Journ.*

---

*How to Deprive Iodine of its Stain.*—Add a few drops of carbolic acid to the tincture, and it will not stain; moreover the tincture is more efficacious, and its action more certain. M. Boggs recommends the following formula for use in injections: Alcoholic tincture iodine, 3 grammes; carbolic acid, 6 drops; glycerine, 30 grammes; distilled water, 150 grammes.—*Ex.*

---

*Salicylic Acid and its Compounds Compared with other Antiseptics.*—According to the results of some comparative experiments which Mr. Lapper has done, the value of salicylic acid in arresting putrefaction is not so high as some other substances which are in use for the same purpose. He obtained some fresh bullock's blood, had it defibrinated, and then mixed it with a one-thousandth part of its weight of various so-called preservative substances; one series of experiments was then conducted at a temperature of 100° F., that of living blood, and the other at the ordinary tempera;ure at which experiments are usually made. Though the amount of the antiseptic employed was small, it was very much larger than is administered in disease, and Mr. Lapper holds the opinion that the usual quantity prescribed is very far from being sufficient to act with special antiseptic force upon such a large mass as the living blood. Salicylic acid in the proportion used was found to have but slight effect in preventing putrefaction. The synthetital acid, or that obtained by Kolbe's process, was slightly superior to the acid from oil of wintergreen. The salicylate of potassium seemed to prevent putrefaction to the same extent as the acid itself. The zinc salt aad an equal potency at blood heat, but a greater at an ordinary temperature. With the zinc sulpho-salicytate it was the reverse, its efficacy being greater at a higher than at a lower temperature. Carbolic acid at the ordinary temperature stood pre-eminent, while at the higher it was inferior in arresting putrefaction to both sulpho-carbolate of zinc and sulphate of quinia. The bisulphite of sodium seemed to hasten putrefaction at 100° F., while at the ordinary temperature it had no effect whatever. Benzoic acid at 100°F. had the same potency as carbolic acid, but at the ordinary temperature it was inferior.—*Dublin Journ. of Med. Science.*—*Med. Record.*

*Nelaton's Method of Resuscitation from Chloroform Narcosis.*—Dr. J. Marion Sims most impressively details his observations respecting the practical workings of the above method. Briefly stated it consists of in reversing the body of the patient, either by hanging him up by the feet or laying him over a bed or table, so that the blood from the greater part of the body can gravitate towards the head. The tongue is drawn forward by a tenaculum and artificial respiration tried. This practice is based upon the theory that death from chloroform takes place from cerebral anæmia.—*Physician's Monitor.*

---

*Death from Ten Grains of Chloral.*—Dr. E. E. F. Ingalls, *Chicago Medical Journal and Examiner,* reports : "A German woman, about thirty-three years of age, apparently healthy, came into the office to have some teeth extracted. She desired some medicine to prevent the pain, and the doctor gave her ten grains of hydrate of chloral, which dose he repeated in one hour. Soon after the second dose the patient manifested alarming symptom of poisoning, and although all was done that could be to resuscitate her, she died in about fifteen minutes. No post mortem examination was made. The patient had taken only two doses of chloral, of ten grains each—the second having been given one hour after the first.—*Ohio Medical Recorder.*

---

*Medical Examinations in Scotland.*—"At the opening of the medical session of Glasgow Uuniversity, last month, Prof. McCall Anderson said few could doubt that a preliminary examination of candidates for admission to the classes was called for, but if proof were required it might be found in the answers given to the following questions submitted to candidates by one of the examining boards: 'What is meant by the antiquity of man? ' Answer : 'The wickedness of man.' 'The letters of Junius?' 'Letters written in the month of June.' 'The Crusades?' 'A war against the Roman Catholics during the last century.' 'The first meridian?' 'The first hour of the day.' 'To speak ironically?' 'To speak about iron.' 'A Gordian knot?' 'The arms of the Gordon family.' 'The Star Chamber?' 'Place for viewing the stars.' 'To sit on the woolsack?' 'To be seated on a sack of wool.' 'A solecism?' 'A book on the sun.' 'The year of jubilee?' 'Leap year.' They could, the Professor added, have appreciated this last answer all the more heartily had it emanated from one of the female medical students. It is however, only just on women to admit that they are, as a rule, serious in their studies, and are not in the habit of joking examiners. There is, indeed an earnestness of purpose in their efforts to compete with man which entitles them to respect, and even imitation."—*Pall Mall Gazette.*

*Chlorate of Potash and Mercurials.*—I notice Dr. J. L. Morrow, of Pittsburg, Pa., asks for information with reference to the use of chlorate of potash with mercurials. Twenty years ago I noticed that bicarbonate of soda combined with calomel and other mercurials prevented salivation; also in a severe case of salivation, if the soda was freely given it quickly cured it. If in a given case I desired to slightly touch the "gams," as we used to call it, as soon as accomplished, I gave soda freely, and three or four days would suffice so remove all trace of mercurial odor.

In after years when chlorate of potash came into general use, my experience satisfied me that it was equally as efficacious as the soda in preventing salivatiou and also in curing it.

I have been practicing medicine over thirty years, and am now and have been using mercurials as freely as when I commenced, and have never had a severe case of salivation since combining soda or chlorate of potash with them.

It would appear that the alkali and mercurials were incompatible, but facts in medicine are stubborn things.

—*Med. and Surg. Reporter.* C. V. Moore, M. D.

---

*Mode of Administering Chloroform.*—In the *Medical Times*, a correspondent who writes from London recommends the following simple form of anæsthetic apparatus:

A pleasant, safe and economical method of administering it will be found in placing a handkerchief in an ordinary tumbler, and then dropping some chloroform upon the handkerchief—the tumbler after this being held to the nose. The advantages of such a plan are these: The tumbler cannot be so applied as to entirely exclude an admixture of air along each side of the nose. Then but a small quantity of chloroform is used, for as soon as each pain is over, the tumbler can be placed bottom upwards on a plate, and the loss by evaporation reduced to a minimum. So slowly does the evaporation take place that the medical attendant can add the chloroform required at intervals himself, and so be sure of the quantity. Further, in emergencies the patient may administer the chloroform herself, the glass falling from the hand as soon as unconsciousness creeps over her. In this respect this plan is much superior to the old one of giving the anæsthetic on the handkerchief alone, which often kept its place after the hand had lost all command over it. As a means of administering chloroform in ordinary cases, the practitioner will find this plan to possess great advantages, and to secure the patient very safely against danger, as well as to be most convenient and economical. Where it is necessary to intrust the administration to a nurse while the medical attendant is engaged otherwise, by this means all risk is avoided, aud occasional supervision is all that is necessary.—*Druggists' Cir.*

*How to Cure "A Cold in the Head."*—Dr. David Ferrier, Assistant Physician King's College Hospital (London,) recommends the use of a snuff composed as follows : Hydrochlorate of morphine, two grains ; acacia powder, two drachms ; trisnitrate of bismuth, six drachms. Of this powder, one quarter to one-half may be taken as snuff in the course of twenty-four hours. The inhalations ought to be commenced as soon as the symptoms of coryza begin to show themselves, and should be used frequently at first, so as to keep the interior of the nostrils constantly well coated. Bismuth alone answers a very good purpose ; but the acacia is added to give greater bulk to the powder, and to make it sufficiently light to be easily snuffed. The morphia is added in cases where it is advisable to strengthen the sedative effect. Dr. Ferrier speaks of it almost as a specific, and as being capable of aborting the cold in a few hours. Our friend Dr. Prout, of Brooklyn, must now look to his laurels.—*Med. Record.*

---

*Death from Self-administration of Chloroform.*—Dr. Gustav Judell, privat-docent and chemical assistant in Professor Leube's clinic at Erlangen, was on October 26 found dead in his bed. He had been accustomed to take chloroform at night as a remedy for sleeplessness, by which he was much troubled ; and a bottle containing the anæsthetic was found near him. It appears that vomiting was excited by the chloroform, but that he was too deeply narcotized to eject the contents of the stomach, so that portions of the food remained in the œsophagus, and caused death by suffocation.—*Brit. Med. Journal.*

---

*An Antidote to Chloroform.*—Dr. Schuller has discovered that the nitrate of amyl quickly removes the effects of chloroform on the vessels of the pia mater, and that even in cases of advanced narcotism from the latter drug, it rapidly relieves the dyspnœa and labored respiration, restoring the strength of the pulse and the reflex excitability. This discovery may prove of much practical value, where chloroform continues to be the favorite anæsthetic.—*Ex.*

---

*Method of Preventing Cloudiness on Exploring Mirrors.*—M. Sanudes, a medical student, communicates to *L'Union Medicale* (Sept. 13) a new process of preventing the laryngeal mirror from becoming obscure. This consists of passing lightly over the mirror a cloth steeped in glycerine. The watery vapour contained in the expired air is dissolved completely in the glycerine and the cloud does not form. He proposes the extension of all glass and optical instruments liable to become obscured by cloudiness.—*Med. Record.*

